# Appendicitis in children: correlation between the surgical and histological diagnosis

**DOI:** 10.1007/s00383-024-05846-2

**Published:** 2024-10-04

**Authors:** Balazs Fadgyas, Georgina Monostori, Dorottya Ori, Peter Vajda

**Affiliations:** 1 Department of Surgery and Traumatology, Heim Pál National Institute of Pediatrics, 86 Üllői Road, Budapest, 1089 Hungary; 2 Division of Pediatric Surgery, Department of Pediatrics, Medical School, University of Pecs, 7 Jozsef Attila Street, Pecs, 7633 Hungary; 3 Department of Pediatrics, Zala County Hospital, Zalaegerszeg, Hungary; 4 Department of Mental Health, Heim Pal National Pediatric Institute, Budapest, Hungary; 5 Institute of Behavioural Sciences, Semmelweis University, Budapest, Hungary

**Keywords:** Children, Appendicitis, Histology, Pediatrics, Pathology

## Abstract

**Purpose:**

Study was designed in order to evaluate the discrepancies between surgical and histological diagnosis in pediatric acute appendicitis (AA) and to compare the outcomes of laparoscopic (LA) and open appendectomies (OA).

**Methods:**

In a retrospective observational cohort, AA patients were included under 18 years of age, operated between 2011 and 2020. Surgical diagnosis was defined by the operating surgeon. The histological findings were classified as uncomplicated and complicated AA. The LOS and complications were also statistically analyzed.

**Results:**

Altogether, 1444 patients were included. Significant strong correlation and a moderate to substantial agreement were found between the surgeon’s and the histopathological findings in all appendectomy cases (weighted kappa value in OA: 0.633, LA: 0.639, total sample: 0.637). If the surgeon’s diagnosis was less severe than the pathologist’s, the LOS was 4 (3;7) days, whereas if the surgical diagnosis was correct, the LOS was 3 (3;5) days (*p* < 0.0001).

**Conclusions:**

In contrary to the literature, our study revealed a strong correlation and moderate agreement between the intraoperative and histopathological findings regarding the severity of AA. Complicated cases are distinctly recognizable during the surgery. In case the surgeon underestimates the severity of AA, the chance of complications is higher.

Level of evidence: II

## Introduction

Acute appendicitis (AA) is the most common surgical emergency in childhood, and its vast majority is treated by appendectomy. The histological results are usually available in 1–2 weeks after the surgery. The postoperative (eg., antibiotic) treatment is usually based on the surgical findings. Little data are available in the current English literature according to the concordance between the surgeon’s opinion (intraoperative findings) and histological results in AA patients. Fallon et al. reported a significant discordance between surgical and pathologic diagnoses [1], on the other hand, Farach et al. found poor agreement between histopathological and surgical diagnosis [2]. In a Brazilian study, Silva et al. found a moderate agreement between surgeon’s and the pathologist’s opinion according to the severity of AA [3]. The intraoperative diagnosis showed strong concordance between the view of the surgeon and the pathologist in children with acute non-complicated appendicitis, and a weak association in complicated cases (gangrenous, ruptured) [4]. In a recent study, agreement in the diagnosis of appendicitis among surgeons and pathologists was found to be weak; however, this was moderate if the cases were classified as perforated or non-perforated [5]. Complicated acute appendicitis (CAA) associated with longer length of hospital stay, higher complication rate (eg. surgical site infection), and readmission than uncomplicated cases [6].

We aimed to study the discrepancies between surgical and histological diagnoses in pediatric appendicitis cases and to compare the outcomes of laparoscopic and open appendectomies. Further goal was to explore the variations in the length of hospitalization and the complication rate when the initial diagnosis of the surgeon was under estimated or less severe compared to the histological diagnosis.

## Materials and methods

Acute appendectomy cases were studied in a retrospective observational cohort. Inclusion criteria were the following: patient’s age 0–18 years operated at our institute between 2011 and 2020, preoperative diagnosis of AA. Patients with malignancies (leukemia, lymphoma, and appendiceal carcinoid) and those with missing data, planned appendectomies (eg., chronic appendicitis, appendectomy following conservative appendicitis treatment) were excluded from the sample. Other parallel infectious diseases (eg., pneumonia, COVID-19 infection, MISC) were not exclusion criteria. The final histological findings were classified as uncomplicated acute appendicitis (UCAA—negative, simplex, catarrhal, phlegmonous, gangrenous) and complicated acute appendicitis (CAA—perforated). The surgical diagnosis was defined by the surgeon who performed the appendectomy, and the histopathological diagnosis by the pathologist. Complicated AA is defined by the surgeon when a visible perforation is observed during the surgical procedure. Laparoscopic (LA), open (OA), also the converted appendectomies were all included, and separately analyzed statistically. Following the diagnosis of AA, the surgeries were performed as promptly as possible, typically on the same day or night. Perioperative antibiotic administration followed the local, institutional protocol. Prior to the appendectomy, a single-dose of co-amoxiclav was administered as a preoperative antibiotic profilaxis. The decision regarding postoperative antibiotic therapy was made by the surgeon. In cases, where pus was present in the periappendicular region (phlegmonous or gangrenous AA), the initial antibiotic could be extended for 24–48 h based on the clinical symptoms (e.g., fever) or elevated inflammatory laboratory parameters (e.g., C-reactive protein, neutrophil ratio, and white blood cell count). In complicated cases (perforated AA), broad-spectrum antibiotic therapy was initiated (e.g., cefotaxime and metronidazole, co-amoxiclav and metronidazole, ceftriaxone and metronidazole, or meropenem) and continued for at least 3–5 days or longer, depending on clinical, radiological (e.g., intra-abdominal abscess) and laboratory results. In case of postoperative intrabdominal abscess, the first chosen antibiotic was meropenem. For CAA cases, intraoperative irrigation with saline solution was performed (1500–2000 ml, depending on patient size). The length of hospital stay (LOS), early (0–1 month after the surgery), and late complications (> 1 month after surgery) were observed. Medical data (operative notes and histological findings) were collected from the electronic patient database (Sanitas X software).

The demographic data are expressed as sample size (n) with a percentage (%). Since our data did not follow the normal distribution, we performed non-parametric tests and displayed interquartile ranges (IQR) when defining age and the length of hospital stay. For correlation measures between the two groups, we used Spearman correlation (Spearman’s rho 0–0.10 as negligible correlation, 0.10–0.39 as weak, 0.40–0.69 as moderate, 0.70–0.89 as strong, and > 0.90 very strong) [7]; whereas for measuring the agreement between the groups, we calculated Cohen’s Kappa values along with 95% confidence intervals and weighted Kappa values that allow disagreements to be weighted differently, which is a useful tool when codes are ordered in measuring interobserver agreement [8] (values 0–0.20 as indicating slight agreement, 0.21–0.40 as fair, 0.41–0.60 as moderate, 0.61–0.80 as substantial, 0.81–1.00 as almost perfect agreement) [9]. For comparison of the two groups, we used the Mann–Whitney *U* test, whereas the Chi-square test was applied to analyze whether there is an association between categorical variables. IBM SPSS 28 (Apache Software Foundation, USA) software was used for the statistical analyses.

## Results

A total of 1444 patients were included in the study. The majority of the patients were male (*n* = 886, 61%), and their median age was 11 years (IQR: 8;14). The intraoperative surgical and histological findings are presented in Table 1.

**Table 1:** Comparison of intraoperative and histopathologic diagnoses of all AA cases

		Surgical diagnoses					
		Negative	Simplex/catarrhal	Phlegmonous	Gangrenous	Perforated	Total
Histological diagnoses	Negative/incipient	29	84	64	1	1	179
	simplex/catarrhal	0	2	5	0	0	7
	Phlegmonous	2	29	856	57	21	965
	Gangrenous	0	1	35	44	26	106
	Perforated	0	0	9	39	139	187
	Total	31	116	969	141	187	1444

Most of the cases were phlegmonous AA (969/1444, 67.1%). The two highest correlations between the surgeon’s and the histopathological findings were found in the phlegmonous (856/965, 88.7%) and the perforated AA (139/187, 74.3%) groups.

A statistically significant strong correlation and a moderate to substantial agreement were found between the surgeon’s findings and histopathological results in all appendectomy cases (Table 2).

**Table 2:** Cohen’s Kappa and weighted Kappa values, and results of correlation between the surgeons’ opinion and the histopathologic findings according to the method of surgery

	Number of cases	Spearman’s rho correlation coefficient	*p*	Cohen’s Kappa with 95% CI	Weighted Kappa	*p*
Open appendectomy	842	0.774	< 0.0001	0.505 (0.460–0.551)	0.633	< 0.0001
Laparoscopic appendectomy	602	0.773	< 0.0001	0.506 (0.449–0.563)	0.639	< 0.0001
Total sample	1444	0.775	< 0.0001	0.506 (0.471–0.542)	0.637	< 0.0001

There was no difference between open and laparoscopic surgery as the 95% confidence intervals of the Cohen’s Kappa values overlapped.

The length of hospital stay was found to be longer in the CAA group than in the UCAA (*p* < 0.0001). Specifically, if the surgeon’s diagnosis was less severe than the pathologist’s, the length of stay was 4 (3;7) days, whereas if the surgical diagnosis was correct, the length of stay was 3 (3;5) days (*p* < 0.0001) (Table 3.).

**Table 3:** Differences in length of hospital stay and complication rate between correct and underrated surgical diagnoses following AA

	Correct surgical diagnose	Underrated AA severity by the surgeon	p
LOS (days)	3 (3;5)	4 (3;7)	< 0.0001
Complication rate	7.9%	18.9%	< 0.0001

If the surgeon underrated the severity of AA, a higher complication rate of 18.9% was found compared to the correct surgical diagnosis, which resulted in a complication rate of only 7.9% (*p* < 0.0001). If the surgeon diagnosed AA as less severe than the pathologist, the chance of complications was found to be higher (OR = 2.693, 95% CI (1.641–4.420)). In addition, the number of early and late complications was higher in CAA patients than in uncomplicated cases (Table 4.).

**Table 4:** Differences between complicated (CAA) and uncomplicated acute appendicitis (UCAA) patients according to the length of hospital stay (LOS) and number of early and late complications

	CAA *n* = 187	UCAA *n* = 1257	p
Median LOS and IQR (days)	8 (7;10)	3 (3;4)	< 0.0001
Early complications	50 (26.7%)	45 (3.6%)	< 0.0001
Late complications	25 (13.4%)	35 (2.8%)	< 0.0001

*IQR* interquartile ranges

## Discussion

The immediate postoperative treatment (eg., antibiotics—single shot vs. wide spectrum) and the length of hospital stay must be planned based according to the surgeon’s opinion. In complicated cases, the length of hospital stay and the number of complications, costs could be higher than in uncomplicated ones [10]. In our study, a strong correlation and a moderate agreement were found between the intraoperative and histopathological findings regarding the severity of appendicitis. Cohen’s Kappa in our research was 0.506, which is similar to Bliss et al. (0.56 and 0.57) [4]. A slight agreement was found by Farach et al. (κ = 0.173) [2] and fair by Fallon et al. (κ = 0.289) [1]. Silva et al. (κ = 0.419) [3], Bliss et al. (κ = 0.56/0.57)) [4] and the current study (κ = 0.506) described a moderate agreement. Substantial agreement was found by Rodrriguez et al. (κ = 0.7)) [5].

Both our study and Fallon et al. used five categories (normal, acute, suppurative, gangrenous, and perforated AA). Bliss et al. applied four (negative, acute, gangrenous and perforated AA), however, Rodriguez, Farach and Silva et al. used only two appendicitis severity groups.

The summary of the agreement between the surgical and histological results of previous studies and the current study is demonstrated in Table 5.

**Table 5:** Overview of the results of different studies

Research group	Length of data collection	Number of patients	Type of the surgical procedure	Appendicitis severity classification	Cohen’s Kappa for the total sample
Bliss et al., (2010) [4]	2 years	133	Open	Normal, acute, gangrenous, perforated	0.57*
Bliss et al., (2010) [4]	2 years	122	Laparoscopic	Normal, acute, gangrenous, perforated	0.56*
Fallon et al. (2015) [1]	1 year	1166	Open and laparoscopic	Normal, acute, suppurative, gangrenous, perforated	0.289
Farach et al. (2015) [2]	1 year	326	Not available	simple or complex	0.173
Rodriguez et al. (2019) [5]	1 year	1092	Open and laparoscopic	Perforated and non-perforated	0.7
Silva (2022) [3]	8 years	485	Open and laparoscopic	Appendicitis with/without necrosis or perforation	0.419
Current study (2024)	10 years	1444	Open and laparoscopic	Negative, simplex, catarrhal, phlegmonous, gangrenous, perforated	0.506

*estimated concordance in the published data

Similar data are available, but to date, our study represents the longest and the largest number of cases on this topic based on the English literature. The advantages of laparoscopic appendectomy compared with the open technique are well described [11–15]. As in the study by Bliss et al., we also did not find a difference between OA and LA groups; the surgeon’s diagnosis was similar in both the groups [4]. Using different appendectomy techniques, the surgeon can well decide the severity of appendicitis and able to plan the postoperative treatment.

The analysis of LOS and the number of complications showed a significant difference between patients with complicated and uncomplicated acute appendicitis. The complication rate was low in UCAA group but high in the CAA group compared to international data [16, 17]. In our study, patients with gangrenous AA were classified within the UCCA group according to the recommendation of Nordin et al. [18]. It is possible that if patients with gangrenous appendicitis had been classified as complicated cases, the complication rate would have been lower in both UCAA and CAA groups. This is because gangrenous AA, which is associated with a higher expected complication rate, represented more severe cases within the UCAA group. Furthermore, in the UCAA group, there would be more severe (gangrenous) appendicitis cases, which may result in higher complication rate. On the other hand, if gangrenous appendicitis would be grouped together as CAA, it would moderate the complication rates in the opposite direction. As in this study, the two highest correlations between the surgeon’s and the histopathological findings were found in the phlegmonous and the perforated histological findings. On the other hand, if the surgeon diagnosed a less severe appendicitis, the length of hospital stay was longer and the complication rate was higher. In these cases, it might be that the antibiotic and supportive therapy was also underdesigned, and when the severe status of the patient was recognized, it was late.

In adult patients, if the surgeon underestimated the histopathologically severity of appendicitis, there was a shorter hospital length of stay and there was no higher rate of readmissions. Holloway JJ et al. suggest that in adults, the intraoperative findings/surgeons’ opinion is enough to plan the adequate postoperative treatment [19]. In children, the appropriate recognition of severe acute appendicitis patients during surgery could be beneficial in several ways. For example, the providers can prepare CAA patients and inform their families/parents about the probable need for a longer hospital stay, and antibiotic treatment can be started on time after the procedure. This can reduce the complication rate and costs as well.

Our study has potential limitations. First, the pathologists were not blinded; the surgical diagnoses could be found in the documentation of the patient. Another limitation of this study is the retrospective design that did not provide us with the advantages of prospective studies (eg. randomization and blinding).

We might conclude that the surgical and histological diagnosis, in one of the largest pediatric hospital in the authors country, strongly correlate and show a high degree of similarity in acute appendicitis cases. Therefore, the postoperative treatment can be well designed and started according to the surgeon’s opinion. In case of surgically “under estimated” patients, the length of hospital stay is longer and the complication rate is higher than in correctly diagnosed patients. These data show the importance of correct intraoperative diagnosis based on a well-planned postoperative therapy.

## Data Availability

No datasets were generated or analysed during the current study.

## Abbreviations

AA: Acute appendicitis
CAA: Complicated acute appendicitis
CI: Confidence intervals
IBD: Inflammatory bowel disease
IQR: Interquartile range
LA: Laparoscopic appendectomy
MISC: Multisystem inflammatory syndrome in children
OA: Open appendectomy
UCAA: Uncomplicated acute appendicitis
UTI: Urinary tract infection
